# Evaluation of the Effect of Glow Discharge Plasma Surface Treatment on Bonding Cements to Zirconia

**DOI:** 10.2174/1874210601812010846

**Published:** 2018-10-25

**Authors:** Abdulelah M. Binmahfooz, Ghadeer I. Basunbul, Aws S ArRejaie

**Affiliations:** 1 Faculty of Dentistry - King Abdulaziz University, Jeddah, Saudi Arabia; 2 Department of Prosthetic Dental Sciences, King Saud University, Riyadh, Saudi Arabia

**Keywords:** Glow discharge plasma, Bonding cement, Zirconia, Cohesive failure, Adhesive failure, Y-TZP ceramics

## Abstract

**Background:**

The major difference in the chemical composition of Y-TZP ceramics, as compared with conventional porcelain, led researchers to develop alternative solutions for achieving durable and long term bonding with the zirconia surface.

**Objective:**

The study aims to evaluate the effects of glow discharge treatment on the bonding between cement and zirconia.

**Methods:**

The zirconia rings and rods were prepared with the Zirconia Y-TZP powder and TZ-3YSB-E (Tosoh-Zirconia) through auto-mix to investigate the glow discharge and thermo-cycling. An orientation Teflon mold was used to centralize each rod into the zirconia ring, and aided as a cementation jig during the cementation procedure.

**Results:**

Cohesive failure (2/3 or more of luting agent remained on the zirconia surface) has been majorly observed with RelyX Ultimate, while adhesion failure (less than 1/3 of the luting agent remained on the zirconia surface) has been primarily observed in Ketac-Cem. Mixed failure was observed among the three specimen including Rely X Unicem 2, Multilink Auto-mix and Ceramir.

**Conclusion:**

The glow discharge surface treatment procedure had a major impact on bond strength to zirconia.

## 1 INTRODUCTION

Replacement of metal-based restorations is a desirable change in dentistry. One of the most promising non-metal-based materials is Yttria-Tetragonal Zirconia Polycrystaline (Y-TZP) ceramics. With the recent demands for maximal esthetics, Y-TZP ceramics are used in every aspect of modern dentistry. Y-TZP ceramics are proven to be a strong and reliable material for dental practitioners, researchers and manufacturers [1-4]. Dental ceramics are based primarily on zirconia that involves a transformation from a tetragonal crystalline phase to a monoclinic phase at the tips of cracks. Such cracks are present in the regions of tensile stress that improve the mechanical properties of dental ceramics [1].

Several techniques have been introduced for improving the strength of a bond related to luting agents along with zirconia surfaces. Basic knowledge of ceramic compositions is required for the application of surface conditioning systems. Amorphuos glass matrix along with the crystalline filler are used for traditional dental ceramics. Unlike Y-TZP ceramics, the intaglio surface of silica-based ceramics are easily roughened by hydrofluoric acid and treated with silane-coupling agent (3-MPS); creating a strong siloxane bond [2, 3]. Lack of a glassy phase and silica in densely sintered zirconia makes it un-etchable; hence, reducing its bond-ability to dental cements [4]. The major difference in the chemical composition of Y-TZP ceramics, as compared with conventional porcelain, led researchers to develop alternative solutions for achieving durable and long term bonding with the zirconia surface [5].

Several bond enhancement techniques are significantly considered with respect to cementation and zirconia surfaces. These techniques include chemical priming [6-11], Phosphate monomer containing luting agents [12-14], Tribo-chemical silica-coating or silicatization [15, 16], airborne particle abrasion along with aluminum oxide particles and Irradiation with Nd:YAG laser.

By using a chemical priming, a chemical bond can be created with the use of modern primers with metacrylate and organophosphate functionalities [6, 7] or bi-functional resin cements [8, 9], containing 10–MDP monomer (10-Methacryloyloxydecyl Di-hydrogen Phosphate) [10]. The phosphate-ester group of MDP reacts with the metal oxides zirconia surface and chemically aids in bonding to zirconia [11]. Several studies have suggested the use of self-etching resin cements for the purpose such as RelyX Unicem (3M ESPE) and Multilink Automix (Ivoclar Vivadent) cements [12-14]. Silicatization involves the development of chemical bonding after the application of kinetic energy as airborne particle along with silica- modified dialuminum trioxide. The application usually provides certain ceramics along with the reactive silica-rich outer surface, which extremely prone to silicatization for the adhesion of resin [15, 16].

Monobond Plus (Ivoclar Vivadent) is a primer that contains 3-Methacryloxypropyltrimethoxysilane (3-MPS). It is known to improve the bond strength to zirconia ceramics [17, 18]. Airborne particle abrasion are aluminum oxide particles that modify with respect to silica. Similarly, it achieves high bond strength from both mechanical and chemical aspects [19]. In addition, YAG laser enhances the surface roughness of Y- TZP ceramics. However, this method longevity is questionable because it creates micro-cracks that can affect the longevity of the restoration.

As per the Intellectual Property Rights (IPR), other manufacturers have developed new phosphate monomers capable of improving the bond to zirconia regarding the structure of MDP-monomer. These include Selective Infiltration Etching (SIE), Glaze-on, and Gluma desensitizer. According to Aboushelib *et al*., [20], SIE is a significant approach, in which zirconia surface can be coated with glass conditioning. It is heated with higher temperature and is used after cooling off to dissolve the glass, revealing the recently formed retentive surface. Similarly, Everson *et al.,* [21] has shown the efficacy of Glaze-on and asserted that it significantly enhances the shear bond strength with resin-based cement when compared with tribo-chemical coating. Another study has shown the potential of Gluma desensitizer, a resin-reinforced layer of dentin, in increasing the shear resin cement’s bond strength to the zirconia surface [22].

However, this study has used glow discharge plasma surface treatment on the bonding between cement and zirconia. Glow discharge plasma surface treatment is an approach, which is used for modifying the physical characteristics without affecting material bulk. This treatment has been previously studied on the shear strength bond [23], supported noble-metal ions [24], and textile dye [25]. According to [23], the preparation of catalyst is effectively attempted through glow discharge plasma as an efficient activation method due to its ability to launch chemical and physical reactions at low temperatures, low power requirement and non-equilibrium properties. Several advantages have been reported, which include selectivity, lifetime, a highly distributed active species, reduced energy requirements, improved catalyst activation, and shortened preparation time. According to [24, 25], glow discharge plasma technique is specifically valuable for surface functionalization because it is possible to change outermost surface layer through this technique. Comparatively, the adhesion properties of the surface are improved through this technique. This treatment negatively charges the material, lowering the surface tension and increasing its surface energy. The action of plasma promotes surface wettability and improves bonding [26-28]. Therefore, this study has aimed to evaluate the effects of glow discharge treatment on the bonding between cement and zirconia. To the best of the researcher’s knowledge, this is the first study to undertake glow discharge treatment on the bonding between cement and zirconia.

## 2 MATERIALS AND METHODS

The *in vitro* design of the study consists of specimen preparation and testing of the hypothesis.

### 2.1 Specimen Preparation

A simulation system has been used comprising of custom-made zirconia rings and cylindrical rods. The zirconia rings and rods were prepared with the Zirconia Y-TZP powder and TZ-3YSB-E (Tosoh-Zirconia) through auto-mix to test the glow discharge and thermo-cycling. Three hundred custom-made zirconia rings were prepared with an outside diameter of 12 mm, height of 6 mm, and a centered hole of 6 mm diameter. Table **1** has presented the formation of cement according to the bonding groups. It has been apparent that RelyX Ultimate, Rely X Unicem 2, and Multilink Auto-mix were bonded through self-etching/self-adhesive groups. However, Ceramir and Ketac-Cem were bonded through acid based reaction. Table **2** presents the specimen distribution for the separation force test with and without glow discharge. A total of “10 glow discharge samples” have been included in group A while “10 No glow discharge samples” are included based on force testing in group B. The zirconia rings were seated in place with a Teflon orientation/cementation jig and all excess cement was removed for groups A and B (Table **2**). All dual-cure resins were polymerized with a Triad 2000 machine (Dentsply, York Division USA) for 60 seconds on each top and bottom surfaces. Then specimens were stored wet at 37 °C for 24 hours. Bonded specimens were placed in a Delrin holder with clearance for the rod inside the holder. The rods were pushed out of the rings with a hardened steel compression rod with a round end, by using a universal testing machine (Instron Model 4202) at a crosshead speed of 0.5 mm / min

Zirconia rings were made by pressing 3 grams zirconia powder (Tosoh–Zirconia TZ-3YSB–E, Lot No. S301047B) with the use of a straight dye set and load of 5000 pounds or 22250N. A putty index has been assembled to enable both the pressing pins provide uniform density independently.

**Fig. (1):**
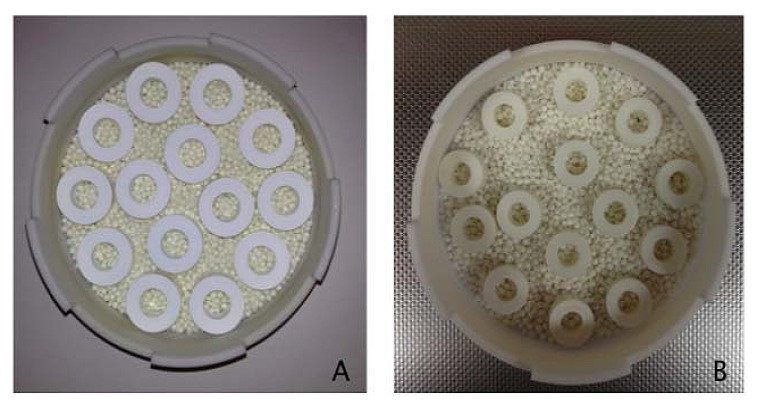
Zirconia rings before and after sintering.

The specimens were first partially fired at 1100°C and then fully sintered in a Vita ZYrcomat furnace (Vident, Brea, CA, USA) at 1530°C, with a holding time of 2 hours. Heating rate has been set at 10°C per minute, as per the manufacturer’s recommendation. After sintering, dimensions of the rings were 12.5 ± 0.1 mm in outer diameter, 5.5 ± 0.1 mm in height and 5.9 ± 0.1 mm in a diameter (Fig. **1**).

Cylindrical shaped grade 2 titanium rods were machined with a uniform diameter of 12.5 ± 0.1 mm and a 5.9 mm height. The diameter of the fully sintered zirconia rings was measured with pin gauges. The titanium rods were machined accordingly, with a cement space of 100 µm. The external surfaces of each rod had airborne particles, which were abraded with 50 µm, average sized aluminum oxide particles, with a pressure of 80 pounds per square inch (6 bars), and at a rate of 4.2 gram per minute. The zirconia rings and the titanium rods were pretreated for bonding according to the manufacturer’s instructions.

An orientation Teflon mold was used to centralize each rod into the zirconia ring, and aided as a cementation jig during the cementation procedure. All cements were mixed as per the instructions, and were applied to the external surface of the titanium rods and the internal surface of the zirconia rings. Peak separation loads were recorded and separation force was measured.

The bond strength was computed as follows:

Bond strength (MPa) = Load (KN × 1000)/ surface area

Where:

Load = Load at separation in KN,

Surface area = 2∏ rh,

r = Radius of specimen (mm),

h = Height of specimen (mm).

### 2.2 Glow Discharge Treatment

Plasma is considered as an ionized gas, which have neutral and charged particles that are known as molecules, electrons, radicals, ions, and atoms. Chamber of glow discharge was evacuated to an optimal pressure by using a vacuum pump (Precision Scientific Co, Chennai, India). Low pressure was maintained by an air inlet. A high frequency voltage (Electro-Technic Products, INC., Chicago, IL, USA) was applied between 2 electrodes, and was also adjusted until stable glow discharge plasma was generated. The schematic and actual glow discharge apparatuses are displayed in Figs. (**2A** and **2B**).

The zirconia rings from group B were placed between two parallel disc electrodes.

### 2.3 Microstructural Examination

Zirconia rings were inspected in three manners:

1. Visual examination.
2. Optical microscopic examination with fiber-optic trans-illumination.
3. Scanning Electron Microscopy (SEM).

Specimens were further treated and inspected under SEM (XL 20, Philips Electronics, Eindhoven, NL, USA) with an acceleration voltage of 15 kV.

### 2.4 Statistical Analysis

Descriptive analyses were recorded as means, standard deviations and coefficients of variance. Furthermore, paired sample t-test has been used to analyze the comparison between adhesive and cohesive groups.

## 3 RESULTS

Failure pattern of specimens displayed a wide range of qualities, including cohesive failure (2/3 or more of luting agent was remained on zirconia surface), adhesive failure (less than 1/3 of the luting agent was remained on zirconia surface), and mixed (adhesive/cohesive failure). The count of the failure modes for the specimens is displayed in Table **3**. As per the cement specimen, cohesive failure has been majorly observed with RelyX Ultimate (n = 12) as compared to 5 specimens of adhesive failure. In addition, adhesion failure has been majorly observed with Ketac-Cem (n = 20) as compared to cohesive failure (n = 0). This indicates that cohesive group was more materialized towards Ketac-Cem cement. Moreover, cohesive failure was also observed with RelyX Unicem 2, and Multilink Auto-mix (n = 8) as compared to adhesion failure (n = 4). This shows that both cements are suitable for adhesive surfaces and proves the applicability of glow discharge treatment for the adhesion surfaces. Table **4** has shown the comparison between adhesive and cohesive failure with respect to cement specimen used in this study. From the findings, it has been clearly observed that there is a significant difference between the use of cement specimens with respect to adhesive and cohesive surfaces.

Selected SEM photographs of each group are displayed in Figs. (**3**-**6**). The observations for failure modes observed during the study are presented in the following figures.

## 4 DISCUSSION

It is commended that the results of this test parameter may not undergo a comparison with other studies. Results further suggested that glow discharge surface treatment procedure had a major impact on bond strength to zirconia. In the current study, 3 self-etching resin cements and 2 acid-base cements were tested using a push-out test under the Instron to measure the separation force.

RelyX Ultimate cement, containing 10-MDP-monomer, recorded the highest separation force values (n = 10), regardless of the surface treatment. It appears that a chemical bond forms between the zirconia metal oxides and the phosphate-ester group of MDP. The bond eventually improves the bonding effectiveness. Such results are similar to those of several studies [7-11, 29-32]. Regarding the Failure mode, the RelyX Ultimate cement group (n = 12) is witnessed with cohesive failure, explaining chemical interaction between MDP-monomer and zirconia surface oxides. Lack of chemical bonding may be rationalized and attributed to the nonappearance of adhesive functional monomers within cement composition as proven in previous studies [11, 33]. Results also indicated that the separation force of Ceramir have been similar to self-adhesive resin cement, Rely X Unicem 2. However, it was significantly higher than the conventional GIC (Ketac-Cem). GIC (Ketac-Cem) separation force values were similar to a study, reported by Jeffreye *et al*., [34]. Ceramirs’ manufacturer (Doxa) has claimed that it has good mechanical properties, which was further confirmed in the present study.

RelyX Unicem 2 and Multilink Automix cements contained Bis-GMA (bisphenol A-glycidyl methacrylate), UDMA (urethane dimethacrylate) or TEGDMA (triethylene glycol dimethacrylate) that have 2 C = C groups at the end of chain. Bonding agent containing MDP (Scotchbond used with RelyX Ultimate, 3M ESPE) has one C = C group. It was hypothesized that the polymerization of MDP-monomer forms a linear polymer; while Bis-GMA and UDMA formed a cross-linked polymer. Cross-linking had a major effect on mechanical properties of polymer [7], which explains the highest separation force gained by RelyX Ultimate. The conventional Bis-GMA cements (Multilink Automix & RelyX Unicem 2) recorded lower bond strengths as compared to RelyX Ultimate. Glow discharge treatment method has improved the surface wettability, which allowed the cements, tested to spread easily.

Studies reviewing different surface treatment techniques on zirconia have presented a contradiction with the findings. It has been reported that surface treatment usually enhances the bonding to zirconia [7-11, 27]. While, it is also suggested that proper cement selection is the major factor that may improve the zirconia bonding [11, 18, 28-31]. However, glow discharge significantly improved the bond strength to zirconia, which appears to be directly related to the increased surface energy and lowered surface tension, caused by the negative charge, created by the glow discharge machine.

It is known that high bond strength can get influenced by the application method of the priming mixture. These systems are thus, perceived sensitive to the technique because the operator tends to have a strong influence on the bonding quality of luting system [35]. In contrast to RelyX Ultimate and Multilink Automix, RelyX Unicem 2 has been observed to be the least influenced by operator as no priming system had been used. Luting systems with no priming agents had a relatively small standard deviation that eventually indicated low sensitivity towards the applied technique. The SEM pictures of RelyX Ultimate and Multilink Automix cements displayed some bubbles incorporation; thus, confirming the technique sensitivity.

## CONCLUSION

The study evaluated the effects of glow discharge plasma surface treatment on the cements that bond to the zirconia. The treatment of glow discharge plasma is a common approach that has been in use for various services in dental health. It has been analyzed that the research lacks proper information about the impact of glow discharge surface treatment on cement bonding to zirconia. It is important to utilize these modes of management with high care as they may lead to the unlikely outcomes, including contamination. Outcomes of this assessment indicated that the glow discharge treatment significantly enhances the retention of zirconia to titanium rods. Thus, the results of the study will be effective in which application area of dentistry.

## Figures and Tables

**Fig. (2):**
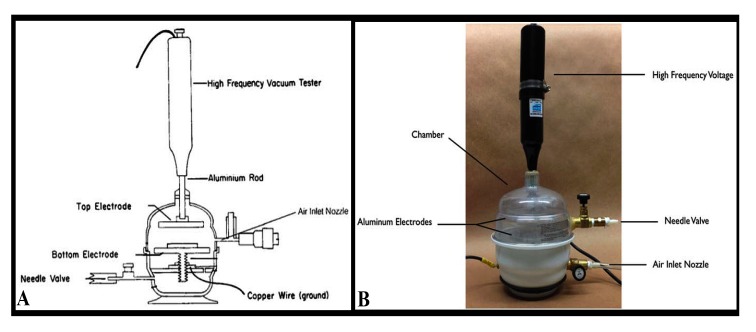
Apparatus of glow discharge machine.

**Fig. (3):**
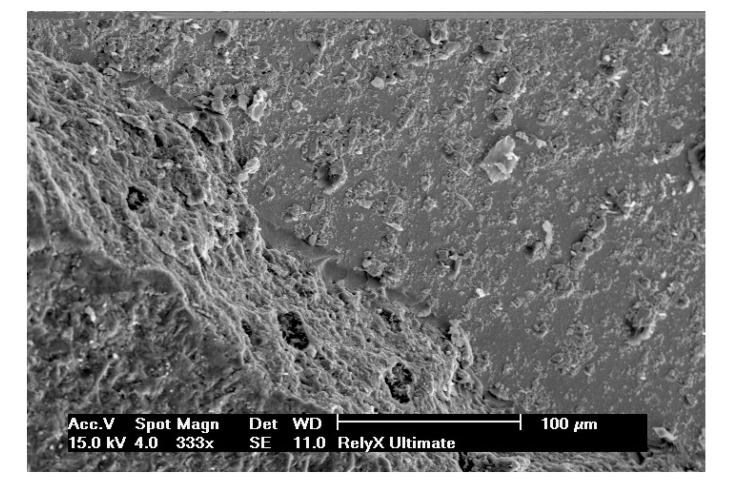
Specimen of RelyX Ultimate glow discharge group displaying a cohesive failure mode (SEM photograph, original magnification 333×).

**Fig. (4):**
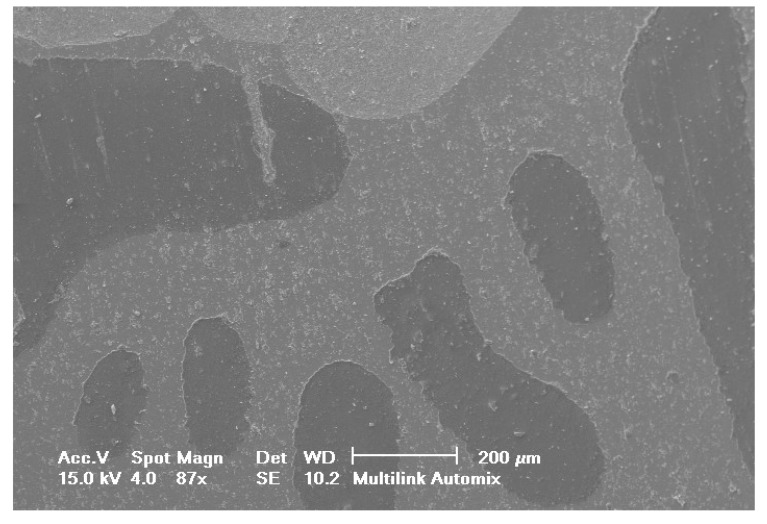
Specimen of Multilink Automix glow discharge group displaying a cohesive failure mode (SEM photograph, original magnification 87×).

**Fig. (5):**
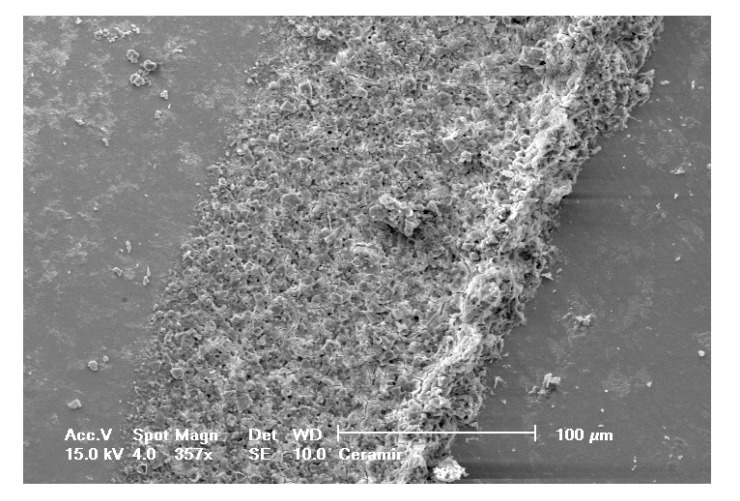
Specimen of Ceramir glow discharge group displaying a mixed failure mode (SEM photograph, original magnification 357×).

**Fig. (6):**
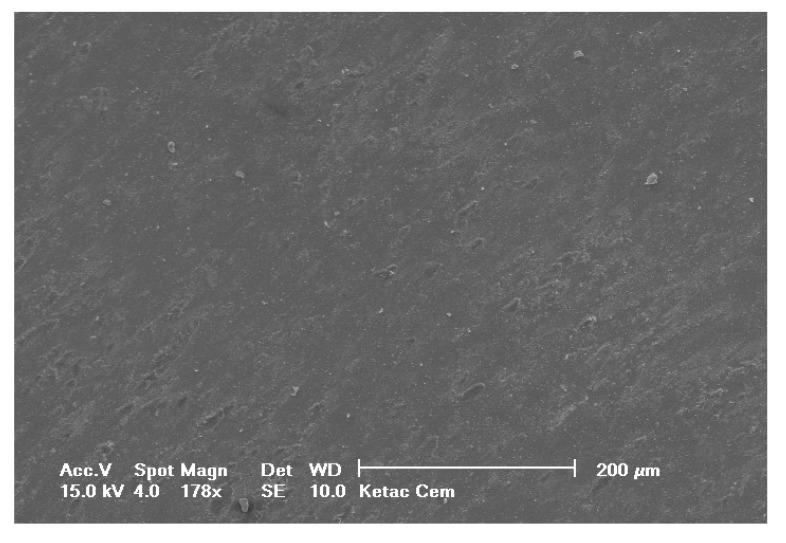
Specimen of Ketac-Cem glow discharge group displaying an adhesive failure mode (SEM photograph, original magnification 178×).

**Table 1:** Classification of Cement Specimen based on Bonding.

**Cement**	**Bonding**	
-	Self-etching/self-adhesive	Acid base reaction
**RelyX Ultimate**	✓	-
**RelyX Unicem 2**	✓	-
**Multilink Auto-mix**	✓	-
**Ceramir**	-	✓
**Ketac-Cem**	-	✓

**Table 2:** Specimen distribution for separation force test.

**Treatment Method**	**Test**	**Sample Size (N)**	**Groups**
**Glow Discharge**	Separation	10	A
**No Glow Discharge**	Force	10	B

**Table 3:** Specimen distribution for the failure mode on glow discharge treatment.

**Cement**	**Failure Mode** **(number of specimen)**		
-	Cohesive	Adhesive	Mixed
**RelyX Ultimate**	12	5	4
**RelyX Unicem 2**	8	4	8
**Multilink Auto-mix**	8	4	8
**Ceramir**	7	5	8
**Ketac-Cem**	0	20	0

**Table 4:** Comparison between Cohesive and Adhesive Groups.

-	**N**	**Correlation**	**Df**	**t-stat**	***P*(T<t)**
**Cohesive**	5	0.88	4	0.9087	2.7764
**Adhesive**	5				
